# The effects of agent hybridization on the efficacy of biological control of tansy ragwort at high elevations

**DOI:** 10.1111/eva.12726

**Published:** 2018-11-27

**Authors:** Marianna Szűcs, Patricia E. Salerno, Brittany J. Teller, Urs Schaffner, Jeffrey L. Littlefield, Ruth A. Hufbauer

**Affiliations:** ^1^ Department of Entomology Michigan State University East Lansing Michigan; ^2^ Department of Bioagricultural Sciences and Pest Management Colorado State University Fort Collins Colorado; ^3^ Department of Biology Colorado State University Fort Collins Colorado; ^4^ Department of Biology Pennsylvania State University State College Pennsylvania; ^5^ Centre for Agriculture and Biosciences International Delémont Switzerland; ^6^ Department of Land Resources and Environmental Sciences Montana State University Bozeman Montana; ^7^ Graduate Degree Program in Ecology Colorado State University Fort Collins Colorado

**Keywords:** classical weed biological control, ddRAD, heterosis, intraspecific hybridization

## Abstract

The success rate of weed biological control programs is difficult to evaluate and the factors affecting it remain poorly understood. One aspect which is still unclear is whether releases of multiple, genetically distinct populations of a biological control agent increase the likelihood of success, either by independent colonization of different environmental niches or by hybridization that may increase the agent's fitness and adaptive ability. Since hybridization is often invoked to explain the success of unintentionally introduced exotic species, hybridization among biocontrol agents may be similarly important in shaping the effectiveness of biological control programs. In this study, we first evaluated intraspecific hybridization among populations of a weed biological control agent, the ragwort flea beetle, *Longitarsus jacobaeae*. These insects were introduced as part of a classical biological control program from Italy and Switzerland. We genotyped 204 individuals from 15 field sites collected in northwest Montana, and an additional 52 individuals that served as references for Italian and Swiss populations. Bayesian analysis of population structure assigned seven populations as pure Swiss and one population as pure Italian, while intraspecific hybrid individuals were detected in seven populations at frequencies of 5%–69%. Subsequently, we conducted a 2‐year exclusion experiment using six sites with Swiss beetles and three with hybrid beetles to evaluate the impact of biological control. We found that biological control by Swiss beetles and by hybrid beetles is effective, increasing mortality of the target plant, *Jacobaea vulgaris*, by 42% and 45%, and reducing fecundity of surviving plants by 44% and 72%, respectively. Beetle densities were higher and mortality of larger plants was higher at sites with hybrids present. These results suggest that hybridization of ragwort flea beetles at high‐elevation sites may improve biological control of tansy ragwort and that intraspecific hybridization of agents could benefit biological control programs.

## 1 INTRODUCTION

Classical biological control, the use of specialized natural enemies from the native range of an invasive species to exert top‐down control, is a crucial component of sound long‐term management of many invasive species. Success rates for classical biological control vary (Schwarzländer, Hinz, Winston, & Day, 2018), and our understanding of what makes some programs more effective than others is limited. Research on existing biological control programs, particularly quantifying the ability of agents to manage their targeted pests, is crucial to understanding the mechanisms behind success or failure. However, measuring the effectiveness of biological control agents in the field presents a considerable challenge; it is expensive and labor‐intensive, and is thus rarely done (Carson, Hovick, Baumert, Bunker, & Pendergast, 2008; Morin et al., 2009; Müller‐Schärer & Schaffner, 2008; Thomas & Reid, 2007). A complication to evaluating effectiveness is that biological control agents themselves are not homogenous populations. In rare cases, cryptic species are inadvertently released, and in others, genetically distinct populations from the native range are released (Bean et al., 2013; Phillips et al., 2008; Winston et al., 2014). The consequences of such diverse releases are generally unknown, including whether distinct biotypes are more likely to establish, whether intra‐ or interspecific hybridization between those occurs in situ (Szűcs, Schwarzländer, & Gaskin, 2011), and whether hybridization may lead to different levels of control in the field.

Hybridization is often invoked as a factor that may contribute to the success of invasive species, as it can have both short‐ and long‐term positive effects on colonizing populations (Colautti & Lau, 2015; Dlugosch, Anderson, Braasch, Cang, & Gillette, 2015; Schierenbeck & Ellstrand, 2009). Since biological control introductions are in essence intentional releases of exotic species in a new range, hybridization might also be important for their success. The consequences of hybridization, however, can be complex, resulting in a range of outcomes from beneficial to neutral or detrimental that may change over time (Arnold & Hodges, 1995; Edmands, 2002).

Hybridization (intraspecific or interspecific) can have immediate positive effects on colonizing populations as early hybrids often show increased fitness (heterosis) due to heterozygote advantage (overdominance), the masking of deleterious alleles, and alleviation of genetic load caused by inbreeding (Lynch, 1991). The effects of heterosis can be potent on colonizing populations as they often pass through a bottleneck that can reduce population size and genetic variation, leading to genetic drift and inbreeding (Dlugosch & Parker, 2008; Estoup et al., 2016; Fauvergue, Vercken, Malausa, & Hufbauer, 2012). The long‐term positive effects of hybridization may come from the increased genetic diversity of hybrids that may facilitate rapid evolution enabling them to adapt faster to novel environments (Arnold, 1997; Stebbins, 1959). In addition, novel recombinants generated by hybridization and subsequent segregation may be better adapted to certain environments than either of their parental species and may therefore be favored by natural selection (Rieseberg et al., 2007; Stebbins, 1959). For example, colonization of extreme habitats by sunflowers was made possible by interspecific hybridization and subsequent selection on extreme or “transgressive” hybrid phenotypes (Rieseberg et al., 2007). While hybrid vigor can be prevalent in the F1 generation, negative effects may manifest in later generations or in backcrosses. In the F2 and later generations, hybrid breakdown or outbreeding depression may occur as a result of previously masked deleterious alleles again becoming homozygous, underdominance (heterozygote disadvantage), or the disruption of coadapted gene complexes (Dobzhansky, 1950; Lynch, 1991; Mayr, 1963). Thus, it is difficult to predict how hybridization may impact fitness. Moreover, little is known whether the heterotic effects or the increased adaptive potential created by hybridization might compromise the host specificity of biological control agents (e.g. Hoffmann, Impson, & Volchansky, 2002, Bitume, Bean, Stahlke, & Hufbauer, 2017).

The few studies that investigated the fitness effects of hybridization in biological control agents found it to be mostly neutral or positive (Bitume et al., 2017; Mathenge et al., 2010; Szűcs, Eigenbrode, Schwarzländer, & Schaffner, 2012), except in the case of crosses between seven different *Trichogramma chilonis* populations, some of which showed outbreeding depression while others showed heterosis (Benvenuto et al., 2012). In terms of host specificity, the effects of hybridization have only been studied between sister species and in species where different populations were adapted to different hosts (Bitume et al., 2017; Goldson, McNeill, & Proffitt, 2003; Hoffmann et al., 2002; Mathenge et al., 2010). In one case, where two populations of the biological control agent *Dactylopius opuntiae* were specific to two different *Opuntia* species, the first‐generation hybrids of these populations were able to attack both hosts but later generations produced both host‐specific and non‐host‐specific genotypes (Hoffmann et al., 2002). The effectiveness of the parasitoid *Microctonus aethiopoides* was also compromised as a biological control agent once populations, specific to different weevil species, admixed (Goldson et al., 2003), and hybridization between three *Diorhabda* species introduced to control invasive *Tamarix* species altered preference in two of three types of crosses toward the nontarget *Tamarix aphylla* (Bitume et al., 2017). These studies indicate that hybridization may have profound effects on the success of biological control by altering fitness and/or host specificity of the biological control agents in the short term. However, since all the above studies were conducted under controlled laboratory conditions using first‐ to third‐generation hybrids, it remains unknown how hybridization impacts biological control in the field and over longer time spans.

Various methods have been used to evaluate the effectiveness of biological control postrelease in the field that can be categorized as correlative, comparative, or manipulative in nature (Morin et al., 2009). The former two approaches rely on either correlating agent densities with damage levels or comparing sites before and after agent release or sites with and without agents (Carson et al., 2008; Denoth & Myers, 2005; Grevstad, 2006; McClay, 1995; Schooler & McEvoy, 2006; Wood & Morris, 2007). While these approaches can provide some indication of effectiveness, they are difficult to interpret due to the correlative nature of the data. Since biotic and abiotic factors change over time and space, even between nearby sites, it is difficult to infer causation. Manipulative experiments, in which agents are excluded with pesticides or cages or included by adding them to cages that cover the target weed in the field, are the most rigorous way to quantify the effects of a biological control agent on its target (Carson et al., 2008; Morin et al., 2009). With these experiments, the contribution of agents to reduction in weed densities or fitness can be directly assessed without confounding effects of abiotic factors (Carson et al., 2008; McClay, 1995). While densities of biological control agents may be unrealistic in inclusion experiments (Briese, Pettit, & Walker, 2004), exclusion experiments can provide a rigorous quantitative assessment of agent effectiveness. Despite their utility, exclusion experiments are rarely conducted postrelease in the field (Catton, Lalonde, Buckley, & De Clerck‐Floate, 2016; Dhileepan, 2003; McEvoy, Cox, & Coombs, 1991; Sheppard & Smyth, 2001; Tipping et al., 2009).

We used a classical weed biological control system to evaluate the extent of intraspecific hybridization between two different agent populations in the field and to measure the effectiveness of biological control provided by parental and hybrid lineages. The weed biological control system includes two genetically distinct populations of the ragwort flea beetle, *Longitarsus jacobaeae* (Waterhouse), one from Italy and one from Switzerland which both were introduced to control the invasive tansy ragwort, *Jacobaea vulgaris* (Gaertn.), in North America. Previous studies revealed that natural hybridization between the Swiss and Italian beetles occurs in the field (Szűcs et al., 2011), and that first‐ and second‐generation hybrids exhibit heterosis in the laboratory in the form of increased fecundity compared to the parental populations (Szűcs, Eigenbrode, et al., 2012). In this study, we first assessed which population(s) of *L. jacobaeae* established in northwestern Montana and to what extent hybridization has occurred in the field. Based on results of molecular analyses evaluating the ancestry of 15 populations, we chose nine field sites, six with pure Swiss beetles and three where hybrids were present, and conducted an exclusion experiment over 2 years to measure plant impacts associated with biological control and to assess whether efficacy is altered by hybridization. We predicted that (a) hybrids would produce more offspring than parental lineages, (b) that hybrids would thus build up higher densities per plant, and hence (c) they would reduce survival and/or reproductive output of the weed more significantly than beetles from parental lineages.

## 2 MATERIALS AND METHODS

### 2.1 Background

Tansy ragwort is native to Eurasia and introduced to North America, where it is prevalent along the Pacific coast from northern California to British Columbia, and has also spread to inland sites in Oregon, Idaho, and Montana (Bain, 1991; Littlefield, Markin, Puliafico, & deMeij, 2008; Szűcs, Eigenbrode, et al., 2012). The plant is a biennial or short‐lived perennial which readily colonizes disturbed areas either via seeds or via vegetative growth from the root crown (Bain, 1991). The plants contain pyrrolizidine alkaloids which render them toxic to cattle and horses (Witte, Ernst, Adam, & Hartmannt, 1992). Long‐term management using mechanical and chemical methods is difficult, due to the persistent, large seed banks and the plant's capacity to regrow after damage such as mowing. Tansy ragwort caused millions of dollars in damage due to livestock poisoning before a biological control program was implemented in the 1960s (Coombs, Radtke, Isaacson, & Snyder, 1996).

The Italian population of the ragwort flea beetle, introduced in 1969 to California, has successfully controlled tansy ragwort along the Pacific coast (Frick & Johnson, 1973; Isaacson, 1978; McEvoy et al., 1991). When ragwort spread to high‐elevation areas in Montana, Italian flea beetles collected from coastal Oregon and from high elevations on Mt. Hood were released from 1997 (Littlefield et al., 2008). Italian beetles in Montana were first released in the Little Wolf Creek (LWC) area at three of the five sites sampled for this study (Table 1). Beetles collected from coastal areas did not establish while those from higher elevations did establish but their populations remained relatively small (Littlefield et al., 2008). To achieve more rapid population buildup, and thus quicker control, a Swiss population of the ragwort flea beetle, preadapted to colder subalpine climates, was introduced to Montana in 2002, both to the LWC area where previously Italian beetles had been released and also to the Island Lake area (Littlefield et al., 2008). An earlier study revealed that by 2007 natural hybridization had occurred between the Swiss and Italian populations at some locations (Szűcs et al., 2011).

**Table 1:** Overview of the 15 *Longitarsus jacobaeae* populations sampled in northwestern Montana in the LWC and Island Lake areas showing the number of individuals genotyped in each population (*N*), the results of assignment test using BAPS 6.0, and the release history of the different populations

Site	Site ID	Location: latitude longitude	Elevation (m)	*N*	BAPS results			Release history	
					% CH	% IT	% Hybrid	Italian	Swiss
1	LWC‐1	48.314	1,063	7	57	0	42.9	None	None
		−114.9506							
2	LWC‐2	48.2889	1,138	14	7.1	64	28.6	**LWC‐2005**	None
		−114.8843							
3	LWC‐3	48.2888	1,172	13	15	15	69.2	None	2002–2005
		−114.8805							
4	LWC‐4	48.284	1,217	14	0	100	0	**2000–2001**	None
		−114.8696							
5	LWC‐5	48.2752	1,312	15	0	87	13.3	**2000–2001**	None
		−114.2752							
6	Island Lake—6	48.2635	1,085	14	100	0	0	None	**2002–2005**
		−114.9617							
7	Island Lake—7	48.2563	1,079	10	100	0	0	2000	**2002–2005**
		−114.9727							
8	Island Lake—8	48.249	1,206	13	100	0	0	None	**2002–2005**
		−114.992							
9	Island Lake—9	48.2362	1,189	10	100	0	0	None	None
		−115.0097							
10	Island Lake—10	48.2323	1,163	16	100	0	0	2000	**2004**
		−115.005							
11	Island Lake—11	48.2212	1,151	13	100	0	0	None	None
		−115.018							
12	Island Lake—12	48.2689	994	19	74	0	26.3	None	None
		−115.0403							
13	Island Lake—13	48.2434	1,262	19	95	0	5.3	**LWC 2011**	**2005**
		−115.0288							
14	Hand Creek—14	48.3252	1,404	12	92	0	8.3	**LWC 2012**	2003
		−114.8003							
15	Island Lake—15	48.249	1,223	15	100	0	0	2000	**2002**–**2005**
		−114.8875							

Notes

Six populations with Swiss and three populations with hybrid individuals highlighted in light gray were used for the exclusion experiment.

Years in bold indicate established populations.

LWC: Little Wolf Creek populations (IT + possible hybrids).

The Swiss and Italian populations of the ragwort flea beetle are morphologically identical, but they have different life histories, which match the climate of their native ranges. The major difference between the life histories of Swiss and Italian beetles is the timing of reproduction and larval development. In Italy where the summers are dry and hot, adult beetles that emerge late spring estivate during the summer and only mate and lay eggs in the fall (Frick & Johnson, 1973). Eggs hatch within 3 weeks and larvae feed on the roots and petioles of tansy ragwort from fall through spring, which weakens the plants and causes high mortality in spring and early summer (Frick & Johnson, 1973). In contrast, Swiss beetles start laying eggs 2 weeks after emergence, in mid‐summer, and remain active during the cooler and moister summers representative in Switzerland (Frick, 1971; Puliafico, Littlefield, Markin, & Schaffner, 2008). Eggs laid during the summer diapause during the winter and larvae emerge and start feeding the following spring (Frick, 1971; Puliafico et al., 2008). Laboratory experiments using first‐ and second‐generation crosses showed that hybrid beetles exhibit intermediate life histories compared to either parent and that they can lay significantly more eggs than their parents (Szűcs, Eigenbrode, et al., 2012). Also, Italian ancestry beetles introduced to Mt. Hood and later to Montana exhibit a shorter summer estival period and faster larval development than low‐elevation Italian populations, likely as an adaptation to the cooler subalpine conditions (Szűcs, Eigenbrode, et al., 2012).

### 2.2 Evaluating the extent of hybridization

**Figure 1:**
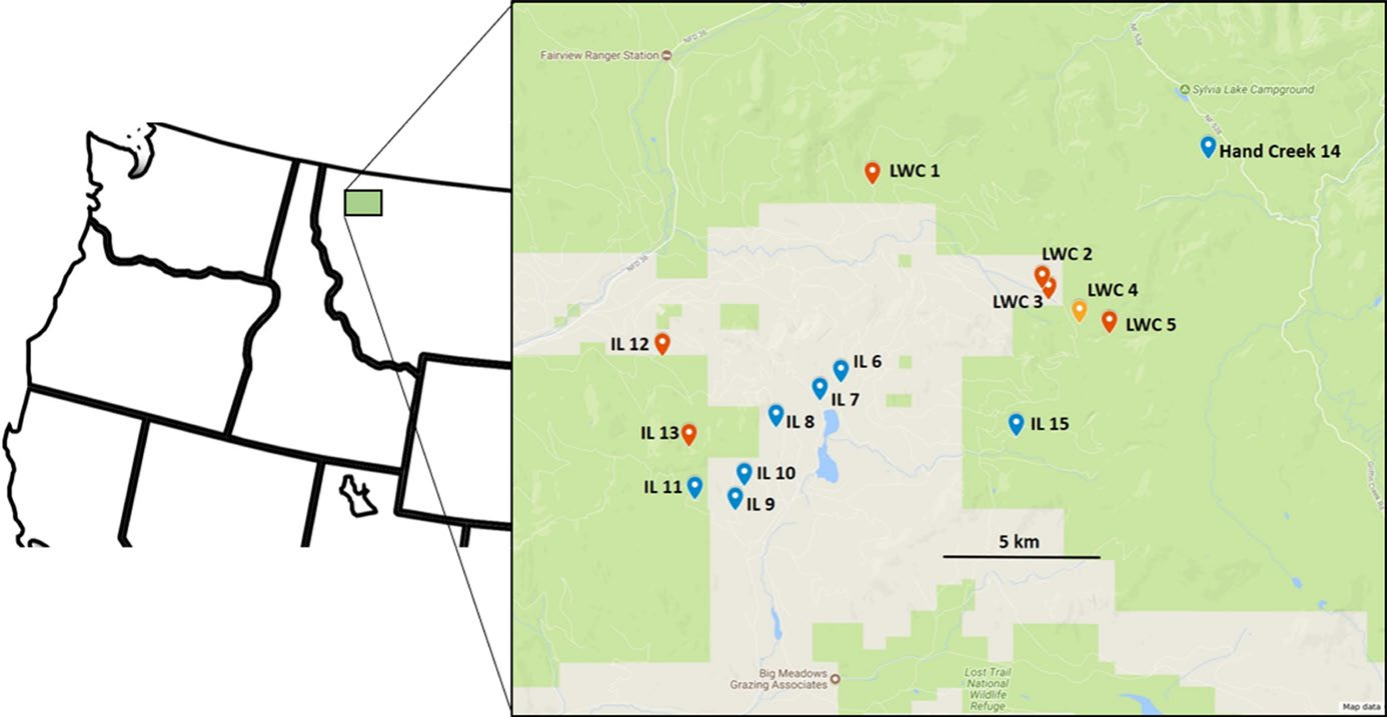
Location of the 15 field sites where *Longitarsus jacobaeae* was sampled and genotyped in northwestern Montana. Populations with pure Swiss individuals are shown in blue, the one population with Italian beetles in yellow, and those that contain hybrid individuals in red. For more information, see Table 1. IL: Island Lake

We assessed the ancestry of *L. jacobaeae* populations by genotyping 7–19 individuals each collected from 15 tansy ragwort populations in Lincoln and Flathead counties in northwest Montana in August and September 2014 (Figure 1, Table 1). The sampling took place in two distinct areas: the LWC drainage (*n* = 5 populations) where the tansy ragwort infestation started and where first Italian and later Swiss beetles had been released; and, the Island Lake area where ragwort spread later and where mostly Swiss beetles had been released (*n* = 9). Beetles from LWC were also released in 2011 at one of the sampled sites in the Island Lake area (Table 1). In addition, a more distant population was also sampled in the Hand Creek area where ragwort also occurred early on resulting in Italian beetle releases initially and Swiss afterward. We also genotyped beetles collected in Switzerland (*n* = 24) and in Salem, OR, (*n* = 28) that served as reference of the Swiss and Italian parental populations, respectively (Szűcs et al., 2011). At each field site, beetles were sampled from multiple plants randomly dispersed throughout the population. We used the double digest restriction‐associated DNA (ddRAD) protocol to generate SNP (single nucleotide polymorphism) markers for genotyping (Peterson, Weber, Kay, Fisher, & Hoekstra, 2012).

DNA was extracted using the DNeasy Blood and Tissue kit (Qiagen) following manufacturer's protocol with two modifications: 4 μl of RNAse A (Qiagen) was added to the lysis step to get RNA‐free genomic DNA, and 50 μl of buffer EB heated to 70°C was used instead of 200 μl of buffer AE for the final elution step. The DNA concentration of each sample was quantified using Qubit Assays (Invitrogen) and then normalized to a standard volume of 200 ng DNA in a total volume of 20 μl. The used ddRAD protocol followed the methods of Peterson et al. (2012) with modifications as per Polato et al. (2017) and multiplexed 288 individuals to be sequenced in a single Illumina 2500 lane. We genotyped individuals using the Stacks v.1.26 (Catchen, Hohenlohe, Bassham, Amores, & Cresko, 2013) pipeline, with default parameters in *process_radtags*. We used ‐*m* 3, ‐*M* 2, and ‐*n* 1 parameters in *denovo_map.pl* and exported the SNP matrix with *populations* with parameters ‐*p* 3, ‐*r* 0.5, and keeping a single SNP per locus. We further filtered the dataset manually by excluding individuals that had more than 10% missing data. The final SNP matrix had 4,058 loci and 256 individuals.

### 2.3 Downstream data analyses

We performed a principal component analysis (PCA) in the R package *adegenet* (Jombart, 2008) to assess general population structure and genomic variance across all populations. We used Bayesian analysis of population structure (BAPS 6.0) (Corander, Marttinen, Sirén, & Tang, 2008) to assess ancestry of sampled individuals. BAPS uses partition‐based Bayesian models to estimate population structure, and we conducted a nonspatial genetic mixture analysis with the “trained clustering” option (Corander & Marttinen, 2006; Corander et al., 2008) which allowed us to use the Italian and Swiss reference populations to predefine known genetic groups to help with assignment of sampled individuals with unknown origin. The ancestry coefficients calculated by BAPS for each individual were visualized using the software Distruct (Rosenberg, 2004).

### 2.4 Evaluating efficacy of parental and hybrid populations

To assess plant impacts associated with different beetle lineages, we used results of population structure from the molecular analyses to select field sites with pure parental and hybrid populations (see Results, Figure 1, Table 1). Sites having low plant density (*n* < 30), including the single site with pure Italian and four sites with hybrid beetles, were excluded. We therefore set up an experiment at six sites where only Swiss beetles were recorded and at three sites where hybrid beetles were present, thus the study is limited to comparing sites with hybrids to sites with one of the parental populations. At each site, 60 randomly chosen tansy ragwort rosettes of various sizes were tagged in mid‐June of 2015, and two treatments were applied. Half of the tagged plants (*n* = 30) were randomly designated as controls at each site and received 1–2 tablespoons of a granular systemic insecticide (Mantra 1G, 1% imidacloprid, NuFarm) depending on plant size to reduce attack by biological control agents. Insecticides were applied upon each monitoring date, that is, monthly between June and September in 2015 and between May and August in 2016. In August 2015, the field sites were inaccessible due to fires in the area, thus at that time no monitoring took place and insecticides were not applied. Insecticide application likely reduced densities of the biological control agent but would not have excluded them entirely. At each monitoring date, plant mortality was assessed, and plant size was measured. For plants in the rosette stage, the longest leaf, while for bolting plants the height of the longest shoot, was measured. In the second year, the fecundity of surviving plants was assessed in August by counting flower heads (capitula) that made up the inflorescence. Beetle densities were estimated by digging up 10 randomly chosen plants from each site (which had not been assigned to either treatment) in June of 2015 and 2016. These plants were measured and then dissected in the laboratory and larvae were counted.

To assess efficacy, we evaluated the influence of treatment (insecticide vs. ambient feeding), beetle ancestry (Swiss vs. hybrid), and their interactions on plant mortality (binary response) using a generalized linear mixed model with a binomial distribution and a logit link function (package “lme4” in R), which included site, and the effects of nesting sites within beetle ancestry as random factors. We compared fecundity of plants that survived and flowered in the second year using a linear mixed model, where treatment, beetle ancestry, and their interaction were fixed effects including Poisson‐distributed errors, and individual‐level random effect to reduce overdispersion, and similar random effects structures to the mortality models. In both cases, we reduced model complexity via backwards model selection (Crawley, 2007), (Supporting Information Tables S1 and S2). Larval counts in June were compared using a linear mixed model, using ancestry and site nested within ancestry and year as random factors. Means and 95% confidence intervals that are presented in the text were estimated based on the data collected in the experiments, but plotted means and 95% confidence intervals or bands were estimated using only the fixed effects of each model. All analyses were performed using R 3.4.3 (R Core & Team, 2013).

## 3 RESULTS

### 3.1 The extent of hybridization

**Figure 2:**
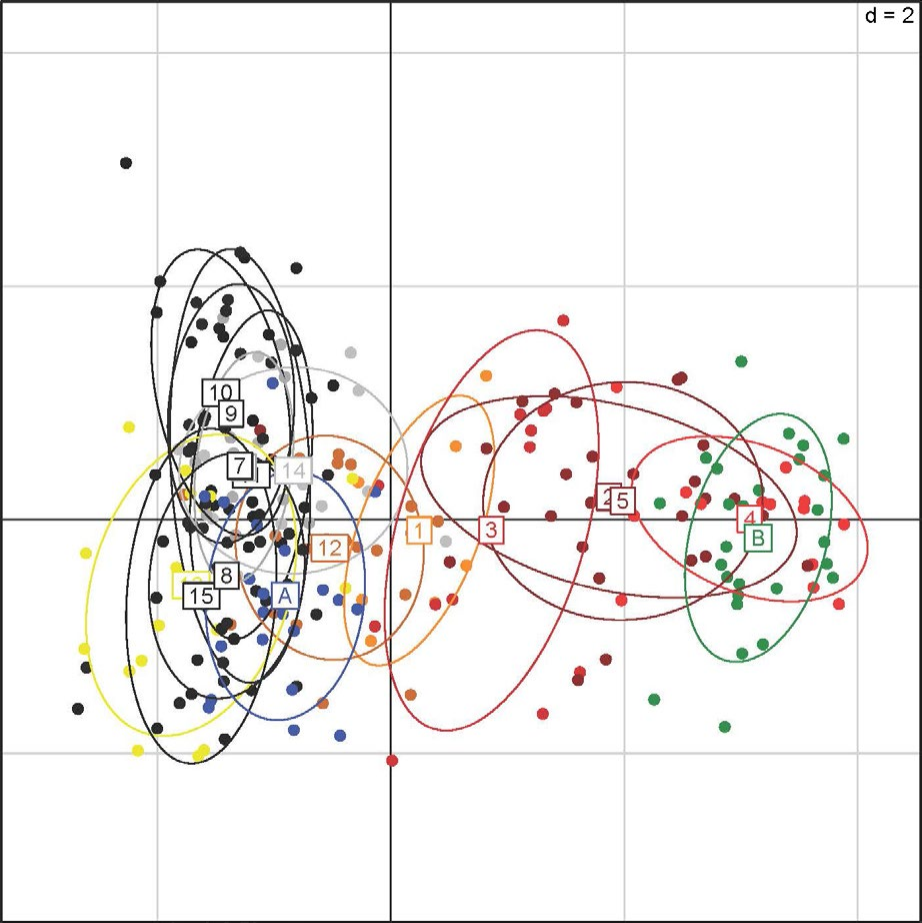
Results of principal component analysis for 15 populations of *Longitarsus jacobaeae* sampled in northwestern Montana and of two reference populations representing the Swiss (blue A) and Italian (green B) parental lineages. Population numbers correspond with those found in Table 1. Populations from the LWC area are shown in shades of red (#1–5), and populations from the Island Lake area that contain only pure Swiss individuals are shown in black (#6–11). Populations from the Island Lake area in which some individuals are assigned a hybrid ancestry based on the results of BAPS are shown in yellow (#13) and orange (#12). The Hand Creek population is shown in gray (#14). Numbers indicate the center point for a given population

The PCA revealed that the first principal component, which contains the highest proportion of genomic variance (3.5%), explains the difference between the two source populations, Swiss and Italian (A and B, respectively; Figure 2, Supporting Information Figure S1). Thus, individuals that are likely hybrids would lie along the axis of variance of the first principal component between the Swiss and Italian populations (Figure 2).

**Figure 3:**
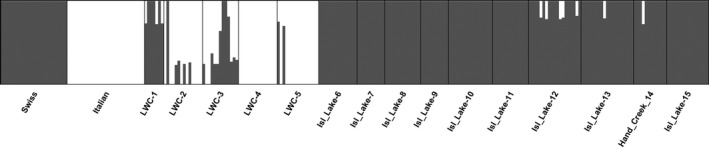
Results of assignment test using genetic admixture analysis in BAPS 6.0. The proportion of Swiss (gray) and Italian (white) ancestry of two reference populations (Swiss and Italian) and 15 populations sampled in northwest Montana are shown. Each bar represents an individual and bars that contain both colors indicate hybrid individuals. See Table 1 for more information on the populations

The admixture analyses in BAPS provided ancestry coefficients for each sampled individual, and its results largely correspond with that of the PCA and release records (Table 1, Figures 2, 3). Populations in the LWC area consist mostly of a mixture of pure Italian (LWC 2, 3, 4, 5) and hybrid individuals (LWC 1, 2, 3, 5) with pure Swiss individuals present only at low frequencies in two populations (LWC 2, 3), and at a relatively higher frequency in one of the populations (LWC 1; Table 1, Figure 3). One population appears to contain pure Italian individuals only (LWC 4). Most populations in the Island Lake area are made up of pure Swiss individuals, except for populations #12 and #13, which contain hybrid individuals in accordance with release records for site #13. Site #12 had no formal releases, but beetles could have dispersed there from site #13 given their proximity (Figure 1). In sum, out of the sampled 15 *L. jacobaeae* populations, one consisted of pure Italian, seven of pure Swiss beetles, and seven contained some proportion of hybrids between the two parental populations (Table 1, Figure 3). In the seven populations where hybridization was detected, hybrid individuals constituted 5%–69% of the sample (Table 1, Figure 2). The largest proportion of hybrids was found in the LWC drainage, where the ragwort infestation started and both Italian and Swiss beetles were released and established early on. Here, hybrids were found in four of the five sampled populations, constituting an average 38.5% of the individuals per population.

### 3.2 Efficacy of parental and hybrid populations

**Figure 4:**
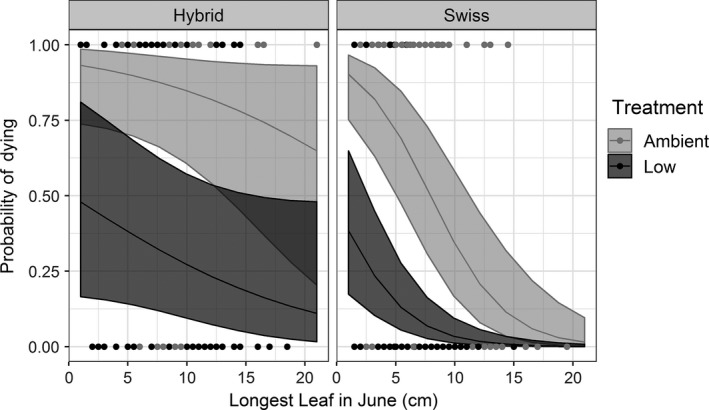
Proportion of tagged plants that died at field sites with either Hybrid or Swiss beetles present when beetles were either allowed to feed normally (ambient feeding—gray line) or were “prevented” from feeding by applying an insecticide to the plants (low feeding—black line). The *x*‐axis shows the size of the longest leaf in cm measured in June 2015 at the beginning of the experiment. Shaded areas represent bootstrapped 95% confidence intervals

Flea beetle feeding significantly increased plant mortality regardless of the ancestry of beetles (*p* « 0.05; Figure 4, Supporting Information Tables S1 and S3). Overall mortality of plants not protected by insecticide was 57% at Swiss and 80% at sites with hybrid beetles but that difference was not statistically significant (ancestry: *p* = 0.8656). Initial plant size, in general, did not influence mortality (*p* = 0.1090). However, large plants were killed at a higher rate at sites where hybrid beetles were present and exerted ambient feeding pressures compared to sites with Swiss beetles (ancestry and initial plant size interaction: *p* = 0.0107; Figure 4).

**Figure 5:**
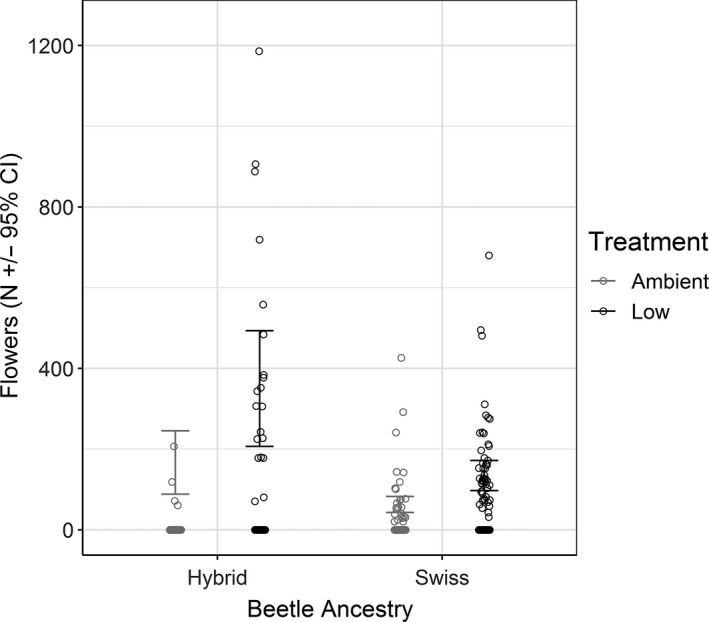
The number of flower heads (capitula) produced by tansy ragwort plants surviving to the second year at field sites where either Swiss or Hybrid beetles were present and when beetles were either allowed to feed normally (ambient feeding—gray) or were “prevented” from feeding by applying an insecticide to the plants (low feeding—black). Bars represent 95% confidence intervals

Plants that survived to the second year and bolted produced fewer flower heads if they were freely attacked by biological control agents than plants that received insecticide that reduced feeding by *L. jacobaeae* (treatment: *p* < 0.0001; Figure 5, Supporting Information Tables S2 and S3). Plants produced more flower heads at sites with hybrid beetles (ancestry: *p* = 0.0006), likely as a result of higher survival of larger plants that were protected by insecticide. The magnitude of flower head reduction by biological control agents was higher at sites with hybrids (72%; CI = 64.2, 130.8) than at sites where only Swiss beetles were present (44%; CI = 37, 34) but that difference was not statistically significant (Figure 5, Supporting Information Table S2).

**Figure 6:**
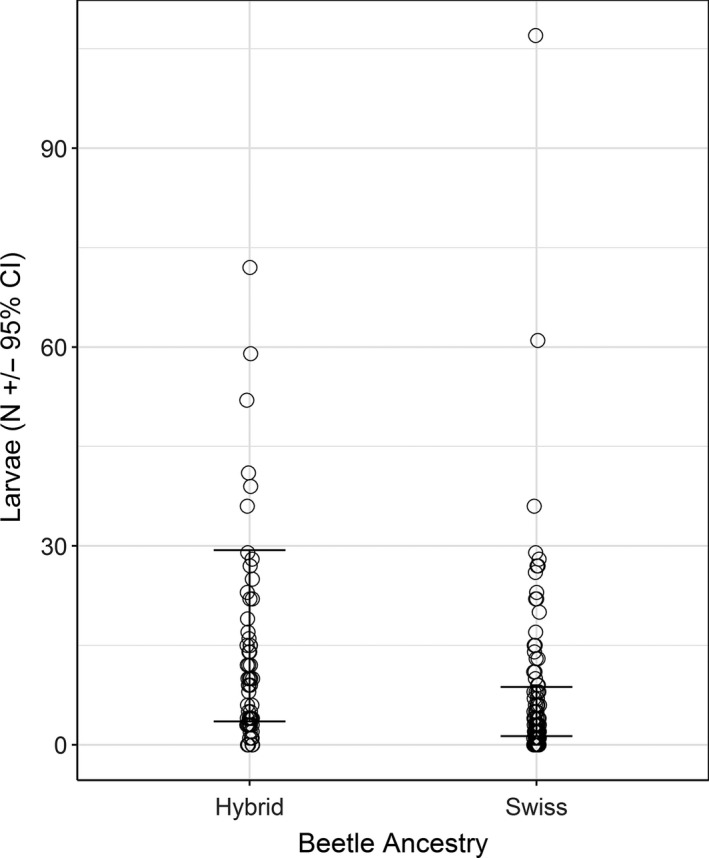
Larval densities at sites where either Hybrid or Swiss beetles were present. Bars represent 95% confidence intervals

Larval densities were higher at sites where hybrids (mean 13.1/plant; 95% CI: 2.4) were present compared to sites with Swiss (mean 6.5/plant; 95% CI: 1.4) beetles (*p* = 0.0089; Figure 6, Supporting Information Table S3). Initial plant sizes did not differ at sites where either Swiss or hybrid beetles were present (*p* = 0.1055).

## 4 DISCUSSION

We found that natural hybridization between Swiss and Italian beetles is widespread in northwest Montana with hybrids being present at most sites where originally both parental populations had been released. Biological control by either Swiss or hybrid beetles is successful, increasing plant mortality by 42% and 45% (Figure 4), and reducing fecundity of surviving plants by 44% and 72%, respectively, when compared to plants treated with insecticide to reduce attack by agents (Figure 5). We found higher larval densities and higher mortality of large plants at sites with hybrid beetles present and no apparent negative effects, which suggest that hybridization in this system has either neutral or positive impacts on biological control.

Genetic analyses revealed that both Swiss and Italian *L. jacobaeae* are established separately in northwestern Montana, have hybridized extensively, and that the hybrids are also spreading through natural dispersal (Figures 1, 2, 3, Table 1). In the LWC area, where the first releases of both Italian and Swiss beetles had taken place, opportunities for hybridization have persisted for about 12 generations. Thus, it is not surprising that almost all sampled sites contained hybrid individuals. It is interesting, however, that pure Italian beetles are still present, making up a relatively high proportion (15%–100%) of given populations (e.g. LWC 2, 4, 5), while very few pure Swiss individuals persist (Table 1). Given that Swiss beetles, and perhaps even hybrids, may be better suited to high‐elevation conditions, one might expect the Italian population to be replaced over time. However, the Italian beetles that established in the area were collected from Mt. Hood, Oregon (Littlefield et al., 2008). A previous study indicated that these beetles have undergone rapid evolution and adapted to the high‐elevation climate at Mt. Hood in less than 30 generations (Szűcs, Schaffner, Price, & Schwarzländer, 2012). Thus, it is likely that Italian beetles are able to persist because of this prior adaptation to high‐elevation climate.

The proportion of hybrid genotypes varied greatly among the sampled sites constituting between 5% and 69% of populations, with hybrids present for at least seven generations at some locations (Szűcs et al., 2011). This indicates that hybrids will likely persist on the long term instead of representing only transient hybrid swarms (Schierenbeck & Ellstrand, 2009). The relative frequency at which hybrids occur in populations is determined by complex interactions between ecological, behavioral, genetic factors and the environment (Arnold, 1997; Stebbins, 1959). The current distribution of hybrids in our populations can be influenced by the number of generations available for outcrossing, which can vary among locations depending on release history and dispersal of both parental and hybrid lineages (Table 1). Since ragwort flea beetles are univoltine, the single generation produced each year allows for relatively slow production of hybrids and/or backcrosses. This may explain why pure parental lineages are still present. It is also possible that despite adaptation of Italian beetles to high elevations, their timing of reproduction with Swiss beetles is not fully synchronized, and hybrids and backcrosses may exhibit a range of reproductive patterns in between the parental lineages. This might result in assortative mating between genotypes with similar timing of reproduction, thus maintaining pure lineages alongside hybrids. However, over time introgression may become so extensive that it swamps out pure parental lineages creating fully admixed populations, as is often the case in intraspecific hybridization (Allendorf, Leary, Spruell, & Wenburg, 2001; Schierenbeck & Ellstrand, 2009; Stebbins, 1959). Given our knowledge of the introduction and hybridization history (Szűcs et al., 2011), resampling of these populations 10–20 years in the future could serve to broaden our knowledge of the long‐term outcomes of hybridization and of the trajectories hybrid swarms may take.

While the consequences of hybridization may vary across taxa, previous studies indicate that in this system the outcomes would likely be neutral or beneficial. In the laboratory, second‐generation hybrids of Italian and Swiss beetles were shown to exhibit heterosis, laying 50%–150% more eggs than either of their parents (Szűcs, Eigenbrode, et al., 2012). Since hybrid vigor is usually strongest in the first generation, these results suggest that hybrids could be equally good or superior biological control agents relative to Swiss beetles on the long term. The higher fecundity of hybrids may result in higher beetle densities, and thus higher attack rates in the field. In partial support of this, we found that larval densities were higher at sites with hybrids than at sites with Swiss beetles. However, we did not find conclusive evidence that the higher beetle densities translated into higher plant mortality or greater reduction of plant fecundity. Even though plant mortality was on average 23% higher and the magnitude of seed reduction 28% higher at sites with hybrid beetles compared to those where only Swiss beetles were present, these differences were not statistically significant. In the context of intentional introduction of species, heterosis was found to increase population growth rates of the weevil, *Callososbruchus maculatus*, in a laboratory experiment (Wagner, Ochocki, Crawford, Compagnoni, & Miller, 2017), and establishment success and persistence of hybrid ring‐necked pheasants in the field (Drake, 2006). While in an invasive freshwater snail, hybrid lineages showed increased invasive potential by outcompeting their parents as a result of immediate heterosis (Facon, Jarne, Pointier, & David, 2005).

Although heterosis could be leading to higher larval densities, another explanation is that hybrid populations may be better adapted to climatic conditions in Montana. The life histories of hybrids tend to be intermediate between the Italian and Swiss parents (Szűcs, Schaffner, et al., 2012). This phenology allows hybrids to start laying eggs during the summer instead of the fall similarly to the Swiss parent, providing them sufficient time to reproduce. In addition, eggs of hybrids can hatch in the fall (M. Szűcs, unpublished data), similarly to eggs of the Italian parent, and thus larvae could feed during the fall and spring, potentially causing more damage to plants than the pure Swiss larvae that only start feeding in the spring. Clearly, hybridization may contribute to increased growth rates and densities of introduced populations by multiple mechanisms but distinguishing between those is often impossible in the field. For example, hybridization has been linked to the evolution of invasiveness in several plant and animal taxa which could be due to the individual or combined effects of heterosis, increased genetic variation, evolutionary novelty, or the alleviation of genetic load caused by hybridization (Schierenbeck & Ellstrand, 2009).

Based on the findings of this study and previous laboratory data on hybrid fitness and phenology, hybridization between Swiss and Italian ragwort flea beetles has either neutral or positive effects on the fitness and impact of this biological control agent. Currently, pure Italian beetles provide good control of tansy ragwort west of the Cascades (Frick & Johnson, 1973; Isaacson, 1978; McEvoy et al., 1991), and we found that Swiss beetles are effective at high‐elevation locations. However, continued spread of ragwort is likely because infestations, for example, in Montana, occur in areas used for recreation and/or grazing, and the long‐lived seeds can easily be transported via vehicles or animals. Ragwort has steadily spread eastward from the west coast to Idaho, Montana, Wyoming, and Colorado (Szűcs et al., 2011; eddmaps.org), and thus, it now grows under a wide range of environmental and climatic conditions. It is unlikely that either the pure Italian or Swiss populations will be optimally adapted to all the different environments. Given this, our management recommendations are to release both the Italian and Swiss ragwort flea beetles in new infestations and let natural selection sort out the best‐fitting genotypes in any given environment. By allowing for hybridization, adaptation may be faster due to the increased genetic diversity of hybrids that selection can act on. However, if hybrids happen to have lower fitness in certain environments, population collapse could likely be avoided because the pure parental genotypes should remain available for selection for several generations since they tend to coexist alongside of hybrids, as shown here.

Besides outbreeding depression reducing fitness, the other concern regarding hybrids in the context of biological control is possible changes to their host specificity. It has been shown that hybridization can alter host specificity in cases where different populations of the same species are specific to different hosts (Goldson et al., 2003; Hoffmann et al., 2002). Hybridization was also found to change preference to different hosts in the case of interspecific hybridization (Bitume et al., 2017). The primary host of both the Italian and Swiss ragwort flea beetles is the target species, *J. vulgaris*, and both populations have a very narrow and similar host range that is limited to the new genus *Jacobaea* and a few species of the old genus *Senecio* (Frick, 1970; Pelser, Nordenstam, Kadereit, Watson, & Watson, 2007; Puliafico, 2003 unpublished). We are not aware of any examples where the specificity of hybrids would change upon crossing two populations of the same species both of which are highly specific to the same host. Thus, it is likely safe to allow for hybridization in this system.

Currently, safety concerns over the possible existence of differences in host specificity of distinct populations have led to tight regulations, requiring host specificity testing of each population of a biological control agent (Barratt, Howarth, Withers, Kean, & Ridley, 2010). This often leads to the introduction of a single population rather than several. However, this change in release strategies may reduce the evolutionary potential of biological control agents to adapt to the biotic and abiotic conditions encountered in the new range. The release of agents from multiple populations may be necessary where, for example, hybridization of the invasive target weed have occurred, and the hybrid weeds are gaining ground in the introduced range. Hybrids of the invasive Brazilian peppertree (*Schinus terebinthifolius*) are the most abundant plants across Florida (Williams, Muchugu, Overholt, & Cuda, 2007). Initial testing of candidate biological control agents has found that one of the agents seems to be highly adapted to specific Brazilian peppertree haplotypes (Cuda et al., 2012). Also, strains of the psyllid *Aphalara itadori*, a biological control agent of Japanese knotweed (*Fallopia japonica*), reveal different performance on the aggressive invader *Fallopia x bohemica*, a hybrid of *F. japonica* and *Fallopia sachalinensis* (Bourchier & Van Hezewijk, 2010). In such cases, a single population of an agent is unlikely to be able to control all parental and hybrid genotypes of the target.

Evidence is accumulating that evolutionary processes can occur within contemporary time scales, which can affect the dynamics of populations (Fussmann, Loreau, & Abrams, 2007; Schoener, 2011; Yoshida, Jones, Ellner, Fussmann, & Hairston, 2003). Hybridization represents an evolutionary pathway that should be considered in the context of biological control as it may impact the performance of both the biological control agents and the target pests. More studies are needed that test both pre‐ and postrelease the effects of hybridization in biological control agents, so guidelines could be developed for when the release of multiple populations and intentional hybridization could be a desirable and safe component of a biological control program.

## CONFLICT OF INTEREST

None declared.

## Data Availability

Data available from the Dryad Digital Repository: https://doi.org/10.5061/dryad.v275qv6.
