# Supplementary material for: The effects of agent hybridization on the efficacy of biological control of tansy ragwort at high elevations
Source: Evol Appl. 2018 Nov 27;12(3):470–81. doi: 10.1111/eva.12726 (PMC6383738; doi:10.1111/eva.12726)
Supplement: Supplementary file 1 [file EVA-12-470-s001.docx]

**Supplementary Tables**

**Table S1.** The model selection process used to analyze mortality (response variable) of tansy ragwort plants in northwestern Montana that were either freely attacked by biological control agents or were treated with insecticide to reduce feeding by biological control agents (Treatment). Six of the 9 field sites (Sites) were populated by biological control agents that originated from Switzerland and at 3 sites hybrid between Swiss and Italian beetles were present (Ancestry). Model 1 was chosen to analyze data on mortality.

| **Model** | **Fixed effects** | **Random effects** | **AIC** | **BIC** |
| --- | --- | --- | --- | --- |
| 1 | Ancestry and Initial size and | Sites nested within ancestry | 483.87 | 513.54 |
|  | Treatment interaction |  |  |  |
| 2 | Ancestry and Initial size interaction | Sites nested within ancestry | 482.45 | 516.36 |
|  | Ancestry and Treatment interaction |  |  |  |
| 3 | Ancestry and Initial size interaction | Sites nested within ancestry | 482.89 | 521.03 |
|  | Initial plant size and Treatment interaction |  |  |  |
|  | Ancestry and Treatment interaction |  |  |  |
| 4 | Ancestry and Initial Size and Treatment interaction | Sites nested within ancestry | 483.53 | 525.92 |

**Table S2.** The model selection process used to analyze differences in fecundity among tansy ragwort plants in northwestern Montana that were either freely attacked by biological control agents or were treated with insecticide to reduce feeding by biological control agents (Treatment). Six of the 9 field sites (Sites) were populated by biological control agents that originated from Switzerland and at 3 sites hybrid between Swiss and Italian beetles were present (Ancestry). Model 1 was chosen to analyze data on fecundity.

| **Model** | **Fixed effects** | **Random effects** | **AIC** | **BIC** |
| --- | --- | --- | --- | --- |
| 1 | Ancestry | Sites nested within Ancestry | 1160.6 | 1176 |
|  | Treatment | Individuals |  |  |
| 2 | Ancestry | Sites nested within Ancestry | 1161.2 | 1179.2 |
|  | Treatment | Individuals |  |  |
|  | Log Initial Plant Size |  |  |  |
| 3 | Ancestry and Treatment interaction | Sites nested within Ancestry | 1164.6 | 1190.3 |
|  | Treatment | Individuals |  |  |
|  | Log Initial Plant Size |  |  |  |
|  | Log Initial Plant Size and Ancestry interaction |  |  |  |
| 4 | Ancestry and Treatment and Log Initial Plant Size interaction | Sites nested within Ancestry | 1165.8 | 1194.2 |
|  |  | Individuals |  |  |

**Table S3**. Results of statistical analyses for plant mortality, plant fecundity, larvae density and initial plant sizes. Parentheses next to the number of observations indicate the number of populations the data represent. Significance codes: 0 ‘***’ 0.001 ‘**’ 0.01 ‘*’

|  | **Fixed effects** | **Estimate** | **Standard Error** | **z value** | **p value** | **Number of observations** |
| --- | --- | --- | --- | --- | --- | --- |
| *Plant Mortality* | intercept | 2.719 | 0.847 | 3.212 | 0.0013** | 512 (9) |
|  | beetle ancestry | -0.170 | 1.003 | -0.169 | 0.8656 |  |
|  | initial plant size | -0.100 | 0.063 | -1.603 | 0.1090 |  |
|  | feeding treatment | -2.701 | 0.280 | -9.650 | < 2e-16*** |  |
|  | beetle ancestry*initial plant size | -0.221 | 0.086 | -2.552 | 0.0107* |  |
|  |  |  |  |  |  |  |
| *Plant Fecundity* | intercept | 4.994 | 0.260 | 19.196 | < 2e-16*** | 97 (8) |
|  | beetle ancestry | -0.903 | 0.260 | -3.449 | 0.0006*** |  |
|  | feeding treatment | 0.773 | 0.160 | 4.846 | 1.26e-06*** |  |
|  |  |  |  |  |  |  |
| *Larvae density* | intercept | 2.317 | 0.542 | 4.278 | 1.89e-05*** | 180 (9) |
|  | ancestry | -1.109 | 0.424 | -2.616 | 0.0089** |  |
|  |  |  |  | *t-value* |  |  |
| *Initial plant size* | intercept | 8.496 | 0.790 | 10.755 | 0.000 | 512 (9) |
|  | ancestry | -1.797 | 0.967 | -1.858 | 0.106 |  |


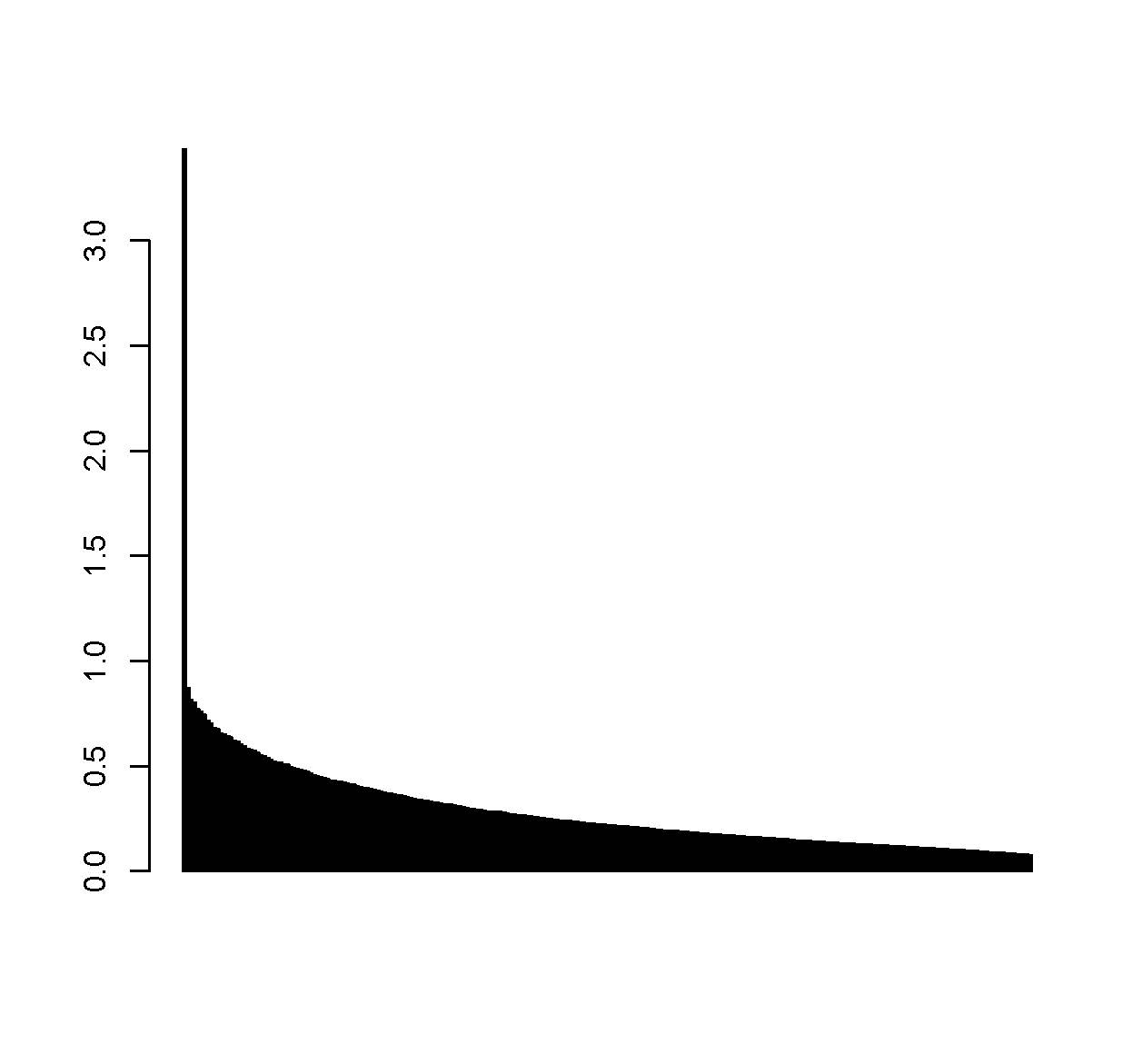
**Figure S1.** Eigenvalues for the principal component analysis shown in Fig. 2 showing the importance of axis 1 for explaining the largest proportion of the genomic variance between the Swiss and Italian populations.
